# LEPREL1 Expression in Human Hepatocellular Carcinoma and Its Suppressor Role on Cell Proliferation

**DOI:** 10.1155/2013/109759

**Published:** 2013-11-11

**Authors:** Jianguo Wang, Xiao Xu, Zhikun Liu, Xuyong Wei, Runzhou Zhuang, Di Lu, Lin Zhou, Haiyang Xie, Shusen Zheng

**Affiliations:** ^1^Division of Hepatobiliary and Pancreatic Surgery, Department of Surgery, First Affiliated Hospital, Zhejiang University School of Medicine, 79 Qingchun Road, Hangzhou 310003, China; ^2^Key Lab of Combined Multi-Organ Transplantation, Ministry of Public Health, First Affiliated Hospital, Zhejiang University School of Medicine, 79 Qingchun Road, Hangzhou 310003, China

## Abstract

*Background*. Hepatocellular carcinoma (HCC) is one of the most aggressive malignancies worldwide. It is characterized by its high invasive and metastatic potential. Leprecan-like 1 (LEPREL1) has been demonstrated to be downregulated in the HCC tissues in previous proteomics studies. The present study is aimed at a new understanding of LEPREL1 function in HCC. *Methods*. Quantitative RT-PCR, immunohistochemical analysis, and western blot analysis were used to evaluate the expression of LEPREL1 between the paired HCC tumor and nontumorous tissues. The biology function of LEPREL1 was investigated by Cell Counting Kit-8 (CCK8) assay and colony formation assay in HepG2 and Bel-7402 cells. *Results*. The levels of LEPREL1 mRNA and protein were significantly lower in the HCC tissues as compared to those of the nontumorous tissues. Reduced LEPREL1 expression was not associated with conventional clinical parameters of HCC. Overexpression of LEPREL1 in HepG2 and Bel-7402 cells inhibited cell proliferation (P < 0.01) and colony formation (P < 0.05). LEPREL1 suppressed tumor cell proliferation through regulation of the cell cycle by downregulation of cyclins. *Conclusions*. Clinical parameters analysis suggested that LEPREL1 was an independent factor in the development of HCC. The biology function experiments showed that LEPREL1 might serve as a potential tumor suppressor gene by inhibiting the HCC cell proliferation.

## 1. Introduction

HCC is one of the most prevalent tumors worldwide and the third leading cause of cancer-related deaths around the world [1, 2]. HCC is characterized by its high invasive and metastatic potential, rapid development, and poor prognosis. Currently, surgical resection, liver transplantation, and radiofrequency ablation have become the three validated curative treatments. However, even after the emergence of those auxiliary approaches, such as RFA or TACE, the 5-year tumor-free survival rate was reported to be approximately 50% with a 5-year tumor recurrence rate of more than 50% after resection [3, 4]. Liver transplantation is widely accepted to be the best method to approach the complete cure of HCC in selected patients. A study suggested that 5-year survival rates of patients fulfilling Milan criteria and patients fulfilling Hangzhou criteria is, respectively, 78.3% and 72.3% [5]. The presence of an unfavorable prognosis is mainly because HCC is a highly vascularized type of tumor with frequent intrahepatic or extrahepatic metastases. In recent years, remarkable progress has been made in the diagnosis and treatment of HCC, but the molecular mechanisms underlying HCC carcinogenesis remains unclear. Understanding the oncogenic role of genetic alteration that might happen during HCC progression is essential for development of innovative therapies. 

Hepatocarcinogenesis is a complex multistep process in which many signaling cascades are altered. The most common mutations include the tumor suppressor gene TP53 (present in about 25–40% of the cancers, depending on the tumor stage) and CTNNB1 gene for **β** catenin (about 25%) [4, 6]. Epigenetic silencing of tumor suppressor genes, including aberrant hypermethylation of CpG islands in promoters and histone modification, is considered a crucial event in the development and progression of tumors [7]. It has been reported that a number of tumor suppressor genes (ASC, CDH1, and RASSF1) are frequently inactivated in HCC [6, 8, 9]. Inactivation of these genes is usually detected with promoter CpG methylation and contribute to the process of carcinogenesis through cell proliferation promotion and apoptosis inhibition [10].

Leprecan-like 1 (LEPREL1) is a protein with an extensive similarity to the Gros1/Leprecan. This protein mainly concentrates in the endoplasmic apparatus and Golgi complex in the cell and is abundant in the basement membranes [11, 12]. Our previous study demonstrated that LEPREL1 was downregulated in the HCC tissues as compared to the adjacent nontumor tissues (data not shown). And LEPREL1 has been reported to suppress the proliferation of the breast cancer cell lines, which potentially makes them rather critical for the cancer diagnosis and treatment [13]. However, previous studies on LEPREL1 were seldom involved HCC. Therefore, this study was arranged to elucidate the correlation between LEPREL1 and HCC with the use of HCC specimens and cancer cell lines.

## 2. Materials and Methods

### 2.1. Patients and Clinical Samples

A total of 80 HCC patients who were treated with hepatectomy in our hospital (First Affiliated Hospital, Zhejiang University School of Medicine, Zhejiang, China) in the second half of 2011 were enrolled in this study. The HCC was preoperatively diagnosed by appropriate imaging characteristics and was verified by histological examination after the operation. The cancer samples and matched noncancerous samples were obtained during the surgery. None of the patients received presurgical chemo- or radiation therapy. Clinicopathologic data were available for each of the 80 patients. Approval for these studies was obtained from the Institutional Review Board of the Faculty of our hospital in accordance with the ethical standards of the responsible committee on human experimentation as well as the declaration of Helsinki.

### 2.2. Cell Culture

Human HCC cell lines (HepG2 and Bel-7402) were obtained from the cell bank of the Chinese Academy of Sciences (Shanghai, China). The cells were cultured in Dulbecco's Modified Eagle's Medium (DMEM) medium (Gibco-BRL), supplemented with 10% fetal bovine serum, in a 37°C incubator with 5% CO_2_.

### 2.3. Total RNA Isolation, Reverse Transcription, and Real-Time PCR

Total RNA was isolated using TRIzol reagent (invitrogen) according to the protocols recommended by the manufacturer. The total RNA concentration and quantity were assessed by absorbance at 260 nm using Nano Drop ND-2000 spectrophotometer. A total of 1 *μ*g of total RNA was used for the first-strand cDNA synthesis with PrimeScript reverse transcriptase (TaKaRa) according to the manufacturer's instructions. Real-time PCR was performed using SYBR Premix PCR kit (TaKaRa) and an ABI PRISM 7500 Sequence Detector. Real-time PCR programs were as follows: one cycle of 95°C for 5 min, 95°C for 5 s, 50°C annealing for 30 s, and 72°C for 34 s, followed by 40 cycles. Three independent experiments were performed for each sample. The relative gene expression levels were determined using the 2-ΔΔCt method. The threshold cycle (CT) was measured during the exponential amplification phase and the amplification plots were analyzed by SDS 1.9.1 software (Applied Biosystems, Foster City, CA). Specific primer pairs were used for LEPREL1 (forward: 5′-ATGTGTGAGGGAACTTGCCACC-3′; reverse: 5′-TTGGCACACTCCAGGGCTTTCA-3′) and GAPDH (forward: 5′-CATCACCATCTTCCAGGAGCG-3′; reverse: 5′-TGACCTTGCCCACAGCCTT-3′).

### 2.4. Western Blot Analysis

12 pairs of HCC samples (100 mg) or cells were lysed in the cold RIPA lysis buffer (Beyotime) supplemented with protease inhibitor phenylmethanesulfonyl fluoride (PMSF). The protein concentration was measured by bicinchoninic acid assay. Protein extracts (40 *μ*g) were separated by 12% SDS-polyacrylamide gel electrophoresis (PAGE) gels (Invitrogen). After electrophoresis, the separated proteins were transferred into polyvinylidene fluoride (PVDF) membranes (Millipore) and were blocked with Tris-buffered saline (TBS) containing 5% nonfat milk for 1 h. The membranes were then incubated overnight at 4°C with the following primary antibodies: rabbit polyclonal LEPREL1(Abcam), rabbit polyclonal GAPDH, rabbit polyclonal Cyclin A2, rabbit polyclonal Cyclin B1, rabbit polyclonal Cyclin D1, rabbit polyclonal Cyclin E2, rabbit polyclonal CDK2, and rabbit polyclonal CDK4 (Epitomics). Primary antibodies were diluted in TBST with 5% nonFAT milk at 1 : 1000. They were then incubated with a secondary goat anti-rabbit IgG monoclonal antibody (Epitomics) conjugated with horseradish peroxidase at 1 : 1000 dilution for 1 h at room temperature. Finally, the protein bands were detected by chemiluminescence using EZ-ECL chemiluminescent detection kit (BIOIND).

### 2.5. Immunohistological Chemistry Staining

The tissue samples of 86 patients with HCC were collected and fixed in 10% formalin before being embedded in paraffin. The tissue sections were cut at 4 *μ*m and were dewaxed in 60°C incubator, followed by an absolute xylene rinse for 10 minutes. The sections were then rehydrated by serially rinsing the slides in 100%, 95%, 85%, and 75% ethanol for 5 min for each concentration. Antigen retrieval was performed by boiling the slides in the antigen retrieval buffer for 20 min, followed by natural cooling. The sections were blocked with 5% FBS-PBS solution for 30 min at 37°C and were incubated at 4°C overnight with rabbit polyclonal LEPREL1 antibody at 1 : 50 dilution. On the next day, the slides were incubated with the secondary antibody for 30 min at 37°C and the DAB was used for staining.

In order to evaluate the immunoreactivity of the LEPREL1 protein, a semiquantitative scoring method was used. The expression level of the LEPREL1-stained cells per field (×200) under microscope was calculated and was compared in different specimens by two separate observers in a double-blind fashion. It was described as a score of 1 (<10% positive cells), 2 (10–20% positive cells), and 3 (>20% positive cells) [14].

### 2.6. Colony Formation Assay

After transfection with pcDNA3.1-LEPREL1 or control vectors for 24 h, the HepG2 and Bel-7402 cells were collected and placed onto the six-well plate (1000 cells per well). After a 14-day growth, the surviving colonies were fixed in methanol, washed two times with phosphate-buffered saline, dried, stained with liquid crystal violet (Sigma-Aldrich, Dorset), and counted. The experiments were repeated in triplicate.

### 2.7. Cell Counting Kit-8 (CCK8) Assay

HepG2 and Bel-7402 cells were plated in 96-well plates at the density of 5,000 cells per well with 100 *μ*L of complete culture medium. After adhesion for 24 hours, the cells were transfected with pcDNA3.1-LEPREL1 or control vectors and were cultured for another 24, 48, 72, and 96 h. The wells where only the culture medium was added in them served as blanks. At each time point, the supernatant was removed and 100 *μ*L of DMEM medium containing 10 *μ*L of CCK8 (Dojindo) was added to each well for another 2 h at 37°C. The absorbance was recorded at 450 nm. All the experiments were independently repeated at least seven times.

### 2.8. Statistical Analysis

The SPSS 10.0 statistical analysis software was used to statistically process the experimental data. The CT values of LEPREL1 in tumor and nontumor samples and the effects of LEPREL1 on cell viability were compared using two-tailed Student's *t*-test. The clinicopathological features of the patients were compared using chi-square test (Fisher's exact test) for categorical variables. A *P* value of less than 0.05 indicated that the differences were statistically significant.

## 3. Result

### 3.1. The Expression of LEPREL1 in the HCC Tissues

**Figure 1:**
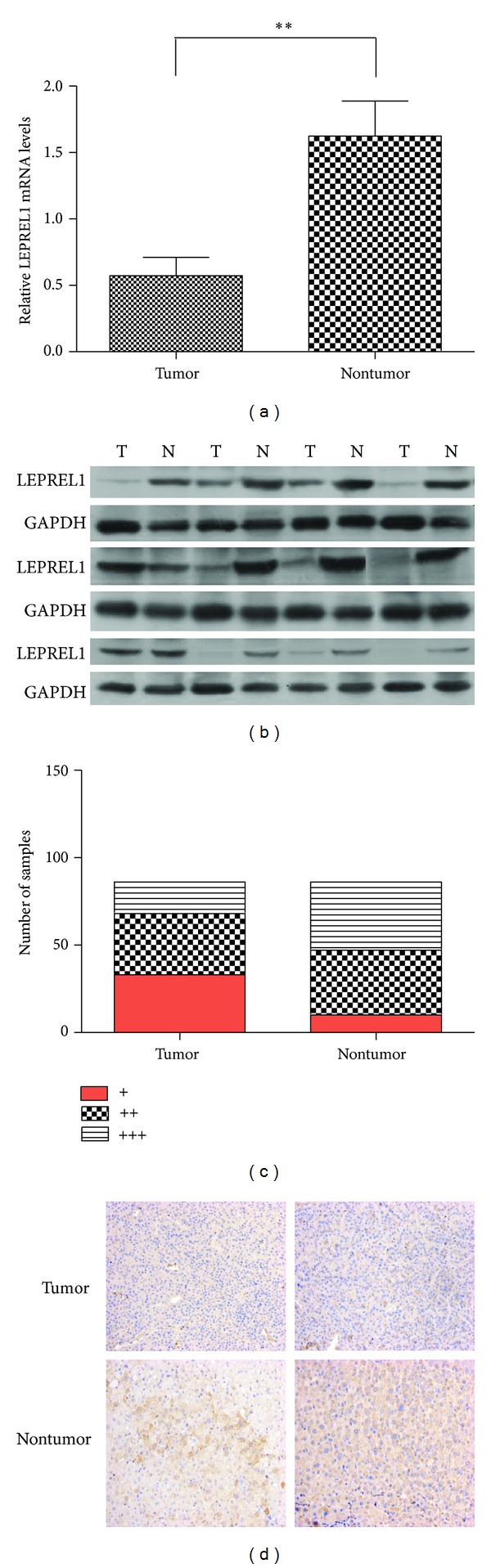
LEPREL1 was frequently downregulated in HCC. (a) The expression level of LEPREL1 relative to GAPDH was compared between the nontumorous and tumor tissues in 80 HCCs using quantitative RT-PCR. Expression of LEPREL1 in tumor tissues was significantly lower than that of the nontumorous tissues (*P* < 0.001, *n* = 80). (b) Representative pictures of LEPREL1 protein expression in randomly selected paired HCC nontumor and tumor tissues. GAPDH was used as an endogenous control. LEPREL1 was downregulated in most of the tumor tissues as compared to the adjacent nontumorous tissues. ((c), (d)) Expression of LEPREL1 in the tumor tissues and adjacent nontumor tissues. Representative pictures of the immunohistochemistry results of LEPREL1 in tissues (d) and histogram of semiquantitatively with a three-tiered system grades (*n* = 86). N: nontumor, T: tumor.

In this study, we first detected the expression level of LEPREL1 using qRT-PCR in 80 pairs of HCC and the matched nontumor tissues. It was noticed that the downregulation of LEPREL1 was detected in 61/80 (76.3%) of the HCC tissues. The CT values of the LEPREL1 in tumor and nontumor tissues were then subjected to the appropriate statistical analysis. The results showed that the expression level of LEPREL1 was obviously lower in the tumor tissues as compared to that of the adjacent nontumor tissues (*P* < 0.001, *n* = 80, Figure 1(a)). The downexpression of LEPREL1 was also observed in the tumor tissues by western blot assay (Figure 1(b)).

The immunohistochemical staining was carried out on 86 HCC specimens and their corresponding adjacent noncancerous livers. As expected, the LEPREL1 expression was significantly lower in 68 (79.07%) of the 86 HCC specimens as compared with that of the adjacent noncancerous livers (Figure 1(c)). The examples of the positive immunostaining for LEPREL1 are shown in Figure 1(d). 

### 3.2. The Correlation between LEPREL1 and Tumor Characteristics

The expression of LEPREL1 was analyzed in HCC with respect to several standard clinic-pathological features (Table 1). However, no significant difference was found between LEPREL1 expression and conventional clinic-pathological features, such as patient age, gender, HBV infection, vascular invasion, histological grade, or AFP level (*P* > 0.05).

### 3.3. Effects of LEPREL1 on Cell Viability

**Figure 2:**
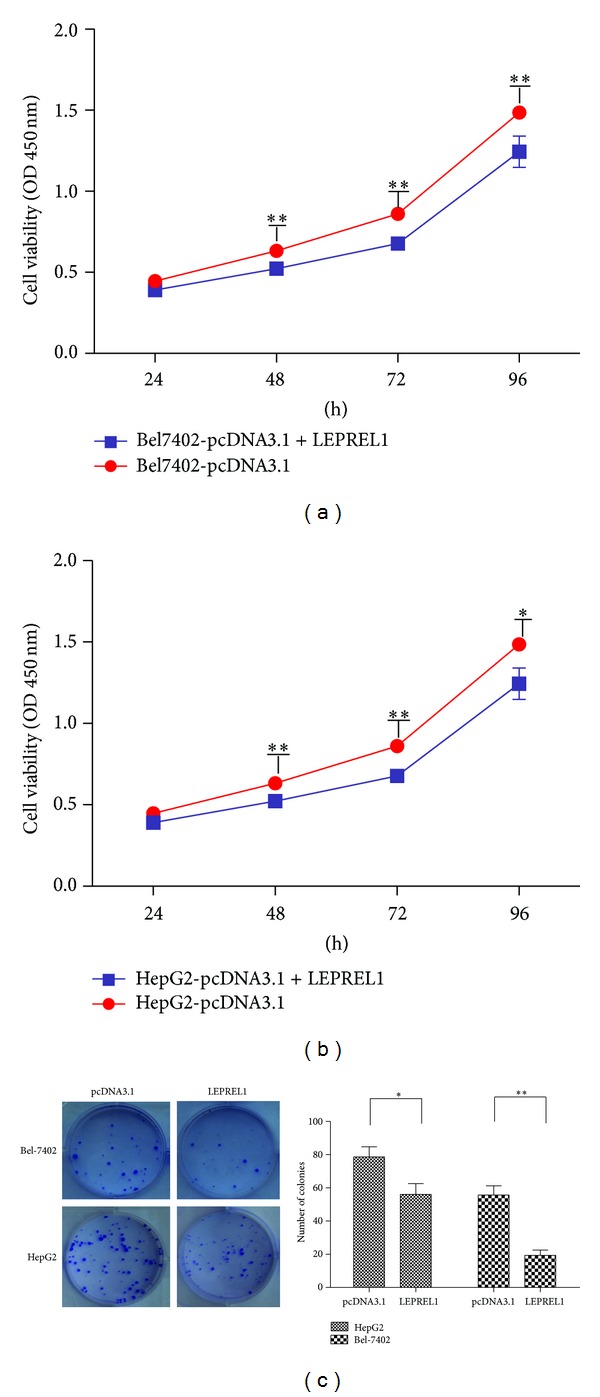
The effect of LEPREL1 on cell growth and colony formation. ((a) and (b)) Exogenous LEPREL1 was expressed in Bel-7402 (a), HepG2 (b) cells transfected with the pcDNA3.1 vector. Parental cells with an empty vector were used as a control. A *t*-test was used to show significant differences between the two groups (*P* < 0.05). (c) To observe the effects of LEPREL1 on colony formation, pcDNA3.1-LEPREL1 was transfected into Bel-7402 and HepG2 cells. Twenty-four hours after the transfection, the cells were plated on the dishes and were cultured in G418 for two weeks. Representative photographs of the colony formation from different stable cell lines are shown in the left panels. The colony formation rate (%) is shown in the right panels (calculated by dividing the colony numbers by 1 × 10^3^ plated cells). The data (mean ± SEM) were obtained from three independent experiments (*P < 0.05; **P < 0.01). The representative dishes showed the inhibitory effects of LEPREL1 on colony formation. The histogram shows that colony formation was significantly suppressed by LEPREL1 as compared with the empty vector control, where the numbers are the mean value of three independent experiments with SD.

**Figure 3:**
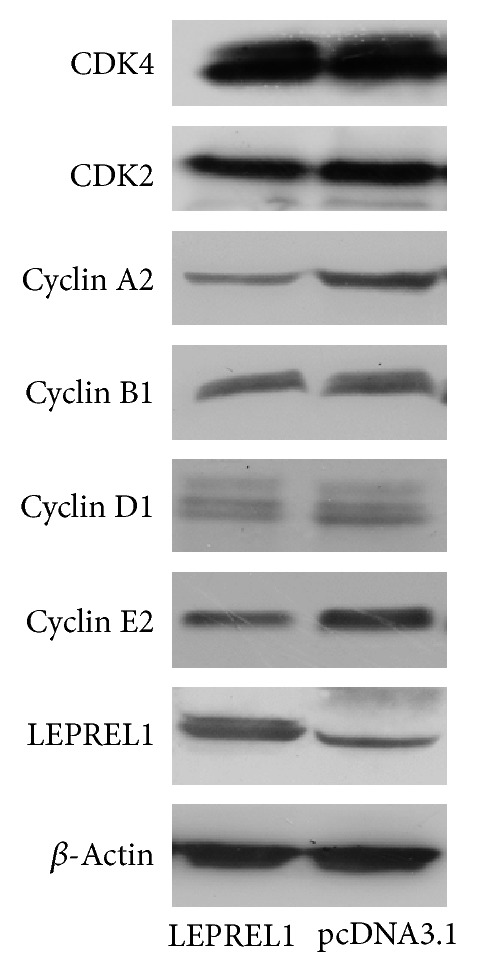
Cell cycle regulatory proteins expression analysis in HepG2 cells treated without (control) and with LEPREL1 for 72 h.

To investigate the tumor suppression ability of LEPREL1 in the HCC cells, we observed the effect of LEPREL1 expression on cell proliferation and colony formation. Cell growth assay showed that the growth of HepG2 and Bel-7402 cells was significantly reduced after the transfection with pcDNA3.1-LEPREL1 as compared to that of the cells transfected with an empty pcDNA3.1 vector (*P* < 0.05, Figure 2(a)). As shown in Figure 3, the frequency of the colony formation in the cells transfected with pcDNA3.1-LEPREL1 was significantly lower than that of the cells with an empty pcDNA3.1 vector. According to our observations in these assays, their ability to suppress the proliferation could imply that LEPREL1 might have the potential to act as a tumor suppressor (Figures 2(b) and 2(c)). 

### 3.4. LEPREL1 Inhibit Cell Proliferation by Modulating Cell Cycle Regulatory Proteins in HepG2 Cells

To understand the potential mechanism of LEPREL1 on inhibiting HCC cell lines proliferation, the expression of major cell cycle regulatory proteins including Cyclins A2, B1, D1, E2, CDK2, and CDK4 was assessed by western blot. The cells transfected with pcDNA3.1-LEPREL1 exhibited a significant decrease in the levels of Cyclin A2 and Cyclin E2 expression when compared with the control but had no detectable effect on Cyclin D1, Cyclin B1, CDK2, and CDK4 expression (Figure 3).

## 4. Discussion

There have been no previous reports describing the LEPREL1 expression patterns in the HCC. In the present study, we found that LEPREL1 was frequently downregulated in HCC. The ectopic expression of LEPREL1 could suppress the proliferation and colony formation, implying that they might play an important role in the HCC progression.

LEPREL1 is a member of prolyl 3-hydroxylases family, which belongs to the family of 2-oxoglutarate dioxygenases. The posttranslational modifications of the collagen including biosynthesis, folding, and assembly are dependent on 2-oxoglutarate dioxygenases. The collagen prolyl 3-hydroxylases catalyze the 3-hydroxylation of different kinds of collagens, which especially occurs in types IV and V collagens [15]. Furthermore, LEPREL1 has been detected in the tissues rich in basement membranes and was reported to participate in the hydroxylation of collagen IV [12]. Type IV collagen along with laminin, perlecan, and nidogen is major components of the basement membrane (BM). The basement membrane is a complex network of interacting proteins, including type IV collagen (Col IV) that acts as a scaffold to stabilize the physical structures of tissues. Type IV collagen also plays an important role in cell adhesion, migration, proliferation, differentiation, and tumor angiogenesis [16]. Moreover, the BM is a physical barrier that prevents tumor invasion. Impaired expression of type IV collagen has been reported to be an early event in the acquisition of an invasive phenotype in some epithelial cancers [17]. Another study has demonstrated that the native Col IV induced an EMT-like process in the MCF10A human mammary nontumorigenic epithelial cells [18]. We speculated that a low expression of P3H2 could affect the properties of the basement membrane, which could facilitate the degradation by the enzyme secreted by tumor cells. There is a need for further investigation to elaborate on the functions of LEPREL1 in cancer invasion.

In addition to the potential functions of LEPREL1 on invasion and metastasis in carcinoma cells, our results demonstrated that LEPREL1 had a direct antiproliferative effect in HCC. Therefore, it was concluded that the implications of this gene might cause tumor suppression. The epigenetic silencing of the tumor suppressor genes could be considered to be a major event contributing to the development and progression of human cancers. Hypermethylation of the DNA cytosine residues at the carbon 5 position (5 mC) in the CpG islands in intragenic, promoter, and intergenic regions is a common epigenetic mechanism in the eukaryotic DNA, which plays an important role during differentiation and in response to some types of physiological changes. Increasing evidence has shown that DNA methylation could be involved in genomic instability and silencing of the tumor suppressor genes in many cancers, including HCC [19–21]. Recently, there has been a number of studies reporting an aberrant methylation of the genes such as GSTP1, RASSF1A, and APC, which has been detected in HCC [22, 23]. The loss of function of these genes as a result of the hypermethylation of the CpG islands in promoters might contribute to the progression of the tumors. It has been reported that the inactivation of LEPREL1 in breast cancer has been attributed to an aberrant CpG methylation in the 5′ regulatory sequence of LEPREL1. Furthermore, the methylation of the LEPREL1 CpG island was specific to the oestrogen receptor-positive breast cancers [13]. The detailed mechanism of the LEPREL1 downregulation in HCC needs further investigation to elucidate whether it could be attributed to the methylation of the CpG islands in the promoter region.

The antiproliferation mechanism of LEPREL1 was further studied by detecting cell cycle regulatory proteins. Overexpression of these cyclins and CDKs altered the cell cycle progression which is closely associated with malignancy. Many studies suggested that overexpression of Cyclins D1, E, and CDK4 protein levels resulting in uncontrolled cell proliferation is closely associated with HCC [24, 25]. Cyclin D1 is a major mitogen-induced regulator of cell cycle progression that has a central function in regulating G1 progression and forms a complex with and functions as a regulatory subunit of CDK4 or CDK6 [26]. Cyclin E/CDK2 complexes have a pivotal role in G1 to S phase transition [27]. Cyclin A2 binds and activates CDC2 or CDK2 kinases and thus promotes both cell cycle G1/S and G2/M transitions. Cyclin B1 expresses predominantly during G2/M phase and forms a cell cycle-dependent complex with p34(cdc2) to promote mitosis [28]. Our results shown that epigenetic expression of LEPREL1 inhibitS the cancer cell proliferation by arresting the G1/S phase via downregulation of cell cycle regulatory proteins, including Cyclin A2 and Cyclin E2.

With a cohort of 86 randomly selected HCC patients, we investigated the potential downregulation of the LEPREL1 with numerous clinical parameters, including age, gender, tumor size, Edmondson grade, vascular invasion, and AFP. However, the expression of the LEPREL1 was not associated with any of the above clinical parameters. 

## 5. Conclusions

This study is the first to examine the role of LEPREL1 in HCC and reveals that the LEPREL1 played a key role in proliferation inhibition of the HCC cell lines. Our results suggested that the LEPREL1 might be a tumor suppressor gene. 

## Figures and Tables

**Table 1:** Clinicopathological characteristic of HCC patients.

	*n *	LEPREL1 score		*P* value
		+++	++~+	
Age				
≦60	56	15	41	0.096
>60	30	3	27	
Gender				
Male	75	14	61	0.231
Female	11	4	7	
HBsAg				
+	64	15	49	0.544
−	22	3	19	
Size of tumor				
≦5	36	4	32	0.066
>5	50	14	36	
Edmondson grade				
I + II	38	9	29	0.603
III + IV	48	9	39	
Vascular invasion				
Without	51	11	40	1.000
With	35	7	28	
Tumor number				
Single	77	14	63	0.087
Multiple	9	4	5	
Liver cirrhosis				
Without	43	12	31	0.184
With	43	6	37	
Coating				
Without	67	15	52	0.751
With	19	3	16	
AFP				
≧400	38	7	31	0.790
<400	48	11	37	

LEPREL1: leprecan-like 1; HBsAg: hepatitis B surface antigen; AFP: alpha fetoprotein.

The significance of the difference between groups in the table was assessed by chi-squared tests (Fisher's exact test).
